# Fingolimod Acutely Facilitates the Activation of TRKB in a BDNF-dependent Manner

**DOI:** 10.1007/s10571-026-01774-y

**Published:** 2026-07-08

**Authors:** Cecilia Anna Brunello, Paula Pastor Muñoz, José Pedro Araujo, Nina Seiffert, Katja Kaurinkoski, Plinio Casarotto, Caroline Biojone, Eero Castrén

**Affiliations:** 1 Neuroscience Center - HiLIFE, University of Helsinki, Helsinki, Finland; 2 Kavli Institute for System Neuroscience, Norwegian University for Science and Technology, Trondheim, Norway; 3 Department of Biomedicine, Translational Neuropsychiatry Unit, Aarhus University, Aarhus, Denmark

**Keywords:** Neuronal plasticity, Allosteric modulators, Neurotrophin, Depression

## Abstract

Fingolimod (FNG) is a sphingosine-1-phosphate receptor agonist currently prescribed for the treatment of remitting-relapsing multiple sclerosis. However, an increasing body of evidence indicates that FNG has a variety of other effects on the central nervous system, making it a good candidate to target other brain disorders that display loss of neuronal cells and synaptic plasticity. FNG treatments induce production of brain-derived neurotrophic factor (BDNF), which promotes neuronal plasticity, arborization and survival via signaling through its cognate receptor TRKB. In this study we characterize the relationship between FNG and TRKB in vitro and in vivo following acute treatments. We found that FNG induces TRKB activation in primary neuronal cultures in a BDNF-dependent way, indicating a rapid effect of FNG. This effect is different from the one elicited by antidepressants and is likely mediated by modulation of plasma membrane properties, as the enhancement of fluoxetine binding and dimerization of the cholesterol-insensitive TRKB mutant Y433F mimic the effects of cholesterol. Moreover, acute FNG treatment normalizes the generalization of conditioned fear response seen in heterozygous BDNF null female mice without affecting the wild-type littermates. Taken together, our data indicate that FNG allosterically promotes TRKB signaling and thereby induces the increase in BDNF production, which mediates the therapeutic effects of the drug on neuronal plasticity.

**Supplementary Information:**

The online version contains supplementary material available at https://doi.org/10.1007/s10571-026-01774-y.

## Introduction

Fingolimod (FNG, FTY720) is a sphingosine-1-phosphate receptors agonist with immunosuppression activity on lymphocytes (Mandala et al. 2002). The compound is currently prescribed to treat relapsing-remitting multiple sclerosis. However, accumulating evidence over the past decade aiming at further elucidating FNG mechanisms of action indicate that the drug has a much wider variety of effects on different cell types and in different organs. In particular, a solid effect of FNG treatment both in vitro and in vivo is the increased level of brain derived neurotrophic factor (BDNF) mRNA and expressed protein in neuronal cells, accompanied by increased spinogenesis and dendritic arborization (Deogracias et al. 2012; Di Pardo et al. 2014; Miguez et al. 2015; Patnaik et al. 2020). Moreover, in animal models of different diseases, FNG rescue motor and cognitive symptoms through increased neuronal plasticity and survival (Deogracias et al. 2012; Kartalou et al. 2020; Miguez et al. 2015; Naegelin et al. 2021; Vidal-Martinez et al. 2019).

BDNF is a neurotrophic factor that, through its cognate receptor neuronal receptor tyrosine kinase 2 (NTRK2, TRKB), modulates synaptic and neuronal plasticity in the brain in an activity-dependent way (Zagrebelsky and Korte 2014). Moreover, BDNF is implicated in the pathophysiology of several brain disorders (Dou et al. 2022). Reduced BDNF levels have been found in brains and serum of severely depressed patients (Dwivedi et al. 2003; Karege et al. 2005), as well as in patients affected by Alzheimer’s, Parkinson’s and Huntington’s disease (Buchman et al. 2016; Huang et al. 2019; Zuccato et al. 2008). In addition, reduction in BDNF-TRKB signaling occurs during chronic stress response (Castrén and Monteggia 2021; Duman and Monteggia 2006; Smith et al. 1995). Taken together, this evidence suggests that imbalances in BDNF-TRKB signaling pathways affect brain functions at different levels and that maintenance or restoration of physiological synaptic plasticity might prevent or ameliorate pathological conditions that manifest with very different symptoms.

While the involvement of BDNF as a therapeutic target of FNG treatment is well established, the molecular mechanisms of BDNF upregulation and promotion of synaptic plasticity remain unclear. TRKB signaling modulates BDNF gene expression in a positive feedback manner (Tuvikene et al. 2016; Yasuda et al. 2007), possibly contributing to the increase of BDNF levels induced by chronic FNG treatment (Deogracias et al. 2012; Doi et al. 2013; Golan et al. 2019; Vidal-Martinez et al. 2019; Yu et al. 2023). ERK signaling and increased BDNF transcription are observed within, respectively, 30 min and one hour following FNG treatment (Deogracias et al. 2012), suggesting that activation of TRKB could be an early event in FNG mechanisms of actions. Additionally, traditional and rapid acting antidepressants also upregulate BDNF production (Chen et al. 2001; Deyama and Duman 2020) and we recently demonstrated that they directly act on TRKB receptors as positive allosteric modulators of BDNF signaling (Casarotto et al. 2021). In particular, antidepressants interact with the cholesterol-sensing domain in the juxtamembrane portion of the transmembrane domain of TRKB (Cannarozzo et al. 2021; Casarotto et al. 2021; Fred et al. 2019), raising the possibility that FNG could also modulate the activity of TRKB in a similar fashion. In this study, we investigate the potential interaction between FNG and TRKB as an early event of FNG-induced plasticity.

## Methods

### Animals

Female adult mice (C57BL/6J-000664, Jackson Labs, maintained in the Laboratory Animal Center of the University of Helsinki), 16–18 weeks old at the beginning of the experiment, carrying a deletion in one of the copies of the Bdnf gene (BDNF.het) or their wild-type littermates were used (Karpova et al. 2011; Laukkanen et al. 2021). The experiments were conducted according to international guidelines for animal experimentation and the County Administrative Board of Southern Finland (ESAVI/38503/2019, ESAVI/40845/2022).

### Drugs

Fingolimod hydrochloride (FNG, 2-Amino-2-[2-(4-octyl-phenyl)-ethyl]-propane-1,3-diol hydrochloride; FTY720, #SML0700, Sigma-Aldrich) was dissolved in DMSO for in vitro experiments and suspended in Tween20 2% in saline for in vivo administration (intraperitoneal, ip; 1 mg/kg volume). In vitro concentrations for FNG were chosen from a concentration range that is considered comparable in terms of brain exposure of the administered drug as well as from previous studies (Foster et al. 2007; Hammarlund-Udenaes et al. 2008). Pravastatin (Orion Pharma) was dissolved in water for oral administration. Fluoxetine (Bosche Scientific #H6995) was biotinylated with EZ Link NHS PEG kit (Thermo scientific). Biotinylated fluoxetine (bFLX), biotinylated BDNF (bBDNF; Alomone Labs, #B-250-B), BDNF (PeproTech, #450-02) and TRKB_FC chimera (R&D biosystems, 688-TK) were dissolved in PBS. PP1 (Sigma-Aldrich, #P0040) was dissolved in DMSO.

### Cell Culture

E18 neuronal primary cortical cultures were prepared as previously described (https://doi.org/10.1042/NS20180207Sahu et al. 2019). Neurons were maintained in neurobasal media (Invitrogen) supplemented with 2% v/v B27 (Invitrogen) and 1% v/v penicillin/streptomycin and L-glutamine (Lonza). Neurons (DIV 10) were pretreated with TRKB_FC (10 µM) or PBS vehicle for 15 min and further treated for 30 min with FNG (0.1, 1 or 10 µM in DMSO) or DMSO vehicle and then processed for ELISA.

Neuronal cell line N2A was used for PCA experiments, while HEK293T cell line was used for the binding assay. Both cell lines were maintained at + 37 °C 5% CO_2_ in DMEM (Lonza) media supplemented with 10% v/v inactivated fetal bovine serum (Invitrogen), 1% v/v L-glutamine and penicillin/streptomycin (Lonza).

### ELISA

Detection of phosphorylated TRKB and interaction with PLCg1 was done with ELISA as previously described (Casarotto et al. 2021; Laukkanen et al. 2021). Briefly, cortical primary neurons were plated 250,000 cells/well on 24 well-plates and treated with FNG (10 µM, 30 min). Cells were washed once with PBS and lysed (20 mM Tris-HCl; 137 mM NaCl; 10% glycerol; 0.05 M NaF; 1% NP-40; 0.05 mM Na3VO4 with protease and phosphatase inhibitors (Sigma). Neuronal lysates were transferred on transparent 96 well-plates previously coated overnight at + 4 °C with total TRKB antibody (R&D, #AF1494) 1:1000 in carbonate buffer and blocked for 2 h with 2% BSA (Sigma) and PBS 0.1% Tween (PBS-T). The plate containing the lysates was transferred back to + 4 °C overnight. Next day, the plate was washed with PBS-T and further incubated overnight at + 4 °C with the biotinylated secondary antibody anti-phosphotyrosine (BioRad; #MCA2472B), anti-phospho Src family Tyr416 (Cell Signaling Technologies; #6943) or anti PLCg1 (Cell Signaling Technologies; #5690) 1:2000 in PBS-T. After a final washing step, the plate was incubated for 2 h at room temperature with HRP-conjugated streptavidin (Thermo Scientific) 1:5000 in PBS-T. Chemiluminescence was measured with a Varioskan plate reader (Thermo Scientific) following the addition of ECL substrate (Thermo Scientific).

### Surface TRKB

Surface TRKB experiments were conducted as previously described (Casarotto et al. 2021; Fred et al. 2019). Briefly, primary cortical neurons were grown 60,000 cells/well in 96 well-plates and treated with FNG (0, 0.1, 1 and 10 µM, 30 min) before being washed with PBS and fixed with 4% paraformaldehyde (PFA) for 20 min at room temperature. PFA was removed and cells were washed three additional times with PBS, then incubated for 1 h at room temperature in blocking buffer without detergents (5% nonfat dry milk, 5% Bovine Serum Albumin in PBS) and left overnight at + 4 °C with the primary antibody anti total TRKB (R&D, #AF1494) in blocking buffer. The next day, the primary antibody was washed away, and cells were incubated with the IgG anti-goat HRP-conjugated antibody for 1 h at room temperature. Finally, following 4 × 10 min washes with PBS, ECL substrate was added and chemiluminescence was measured with a Varioskan plate reader (Thermo Scientific).

### Protein-Fragment Complementation Assay (PCA)

PCA is based on a humanized *Gaussia princeps* luciferase system originally described in Prof. Michnick laboratory (Remy and Michnick 2006). Specifically, split and complementary portions of the luciferase were cloned on the C-terminus of the rat TRKB receptor in pcDNA3.1/zeo backbones. Interaction of the overexpressed proteins of interest, here two TRKB molecules, allows the reporter luciferase to refold in its original and functional conformation and to produce light as PCA signal, following the addition of its substrate native coelenterazine, in a proportional amount of interacting TRKB molecules at that given time.

PCA experiments were conducted as already described (Brunello et al. 2016; Merezhko et al. 2018). Briefly, 10.000 N2A cells/well were seeded in white-walled 96 well plates and transfected 24 h after plating with the phGLuc-TRKB plasmids (110 ng of DNA per well) with Lipofectamine 2000 (Invitrogen) according to manufacturer’s instructions. Transfected cells were incubated for an additional 24 h. FNG treatments were performed 30 min before measurements and culture media containing the treatments was changed to phenol red-free and serum-free DMEM (Invitrogen). PCA signal was measured as luminescence with a Varioskan plate reader (Thermo Scientific) immediately after the injection of the substrate for the *G. princeps* luciferase, native coelenterazine (25 µM, NanoLight Technology).

### Ligand Binding Assay

The cell-free binding assay was performed as previously described (Cannarozzo et al. 2021; Casarotto et al. 2021). Briefly, HEK293T cells transfected with TRKB.wt construct were lysed (see ELISA section for details) and transferred to transparent 96-wells plates (HB, Perking Elmer) previously coated overnight with TRKB antibody (1:1000 in carbonate buffer, + 4 °C) and blocked with 3% BSA in PBS for 2 h at room temperature. The samples were incubated overnight on the shaker at + 4 °C. The following day, after 3 × 10 min PBS washes, biotinylated BDNF (0, 2, 4, 6, 8, 10 ng/ml, Alomone Labs, #B-250-B) was added with or without FNG (0, 0.1, 1, 10 µM) for 1 h at room temperature. For the competition binding assay, a fixed concentration of Fluoxetine (1 µM) was incubated with increasing concentration of FNG (0, 0.01, 0.1, 1, 10, 100 µM). After washing away the treatments, the samples were incubated with HRP-conjugated streptavidin (1:10000, Thermo Scientific) for 1 h at room temperature. Luminescence was measured with a Varioskan plate reader (Thermo Scientific) after the addition of ECL substrate according to manufacturer’s instructions (Thermo Scientific).

### qPCR

N2a cells were tested for the endogenous expression of TRKB. As a control, cells were transfected with a TRKB.wt plasmid as described in the previous section. Primers for assessing levels of TRKB mRNA following FNG treatment were synthetized by Sigma-Aldrich. TRKB primers were: forward, GAAGGGAAGTCTGTGACCATTT-3′, reverse: 5′-GTGTGTGGCTTGTTTCATTCAT-3′. Beta actin was used as housekeeping gene: TGTCACCAACTGGGACGATA-3′, reverse: 5′-GGGGTGTTGAAGGTCTCAAA-3´. RNA isolation from lysates was performed with PureLink RNA Mini kit (Thermo Scientific), while reverse transcription of RNA was carried out with LunaScript RT SuperMix Kit (New England Biolabs), according to manufacturer´s instructions. SYBR Green Maxima kit (Thermo Scientific) and BioRad CFX Opus 96 PCR cycler were used to detect relative mRNA amounts. Analysis was performed using the 2^ΔΔCT^ method in Microsoft Excel.

### Spinogenesis

Spine formation following FNG treatment was performed as previously described (Brunello et al. 2026). Briefly, primary hippocampal neurons were seeded at a density of 50.000 cells per well on coverglasses until 21 days in vitro (DIV21). Cells were treated with FNG (1 µM, 24 h) and BDNF as positive control (10ng/ml) and fixed with 4% paraformaldehyde (PFA) for 20 min at room temperature. Cells were washed three times with PBS and blocked for 1 h at room temprature in blocking buffer (10% normal donkey serum, 1% bovine serum albumin (BSA) and 0.1% gelatin, 0.1% Triton X100 and 0.05% Tween-20). Primary antibody MAP-2 (Invitrogen, PA5-17646) was incubated overnight at 4 °C diluted 1:1.000 in blocking buffer, without detergent.

Cells were washed three times with PBS and incubated with Alexa Fluor 488-conjugated anti-rabbit secondary antibody (Invitrogen, 1:1000) for 1 h at room temperature. Following washing, F-actin staining was performed using phalloidin (#A22287, Invitrogen,1:40 in PBS) for 1.5 h at room temperature, and washed with 0.1 M phosphate buffer before mounting on glass slides with Dako mounting medium (#S3023, Agilent). Images were acquired using a Zeiss LSM 980 confocal microscope and a plan-apochromat 63x/1.4 water objective. Each condition included 3 separate coverslips; for each coverslip, five neurons were imaged, and two 15 μm regions were selected from secondary or tertiary dendrites per neuron were analyzed. The number of spines was manually counted using ImageJ in a blinded setting.

### Contextual Discrimination Task

The paradigm used for contextual fear conditioning was adapted from a previous study (Laukkanen et al. 2021). We used 25 animals between BDNF.hets and their wild-type littermates, and 5 animals per group for the pravastatin cohort. Pravastatin (10 mg/kg) was administered for 14 consecutive days in drinking water in a non-supervised manner (Casarotto et al. 2021). The animals were anesthetized with isoflurane (2% for 4 min) and received either vehicle or FNG (1 mg/kg) by intraperitoneal injection twice during the experiment: 2 h before the conditioning and 2 h before the exposure to the unfamiliar context. The selected mouse dose was based on human equivalent dose calculations using body surface area normalization and supported by prior preclinical studies (Nair and Jacob 2016). No behavioral acute effects of the vehicle, nor discomfort or irritation at the site of injection was noticeable. The conditioning was performed in context A (transparent walls 23 × 23 × 35 cm with metal grid bottom) for a total of 10 min, including a short habituation, the foot-shock session (0.6 mA/2 seconds, at intervals of 30 s/1 minute) and the last 5 min of recording of the freezing behavior of the animals. After 24 h, animals were placed in the unfamiliar context B (box of same dimensions of context A but with black walls and black sleek bottom, no foot shocks) for 5 min. After 24 h, animals were returned to context A for 5 min, without receiving any shock. Animal freezing (measured in seconds) was recorded with the TSE software (Bad Homburg, Germany).

#### Statistical Analysis

The data was analyzed in Prism Graphpad 8.1.1 by Student’s t-test (two tails), one-, two and three- way ANOVA or Kruskal-Wallis as non-parametric tests, followed by Tukey and Dunnett’s post hoc tests. For the in vivo experiment, data points refer to single animal, while for the in vitro experiments data points refer to single culture. In vitro experiments were repeated 2–3 times each. Replica numbers as well as specific p-values are indicated in figure legends.

## Results

### FNG Promotes TRKB Dimerization and Signaling In Vitro

**Fig. 1:**
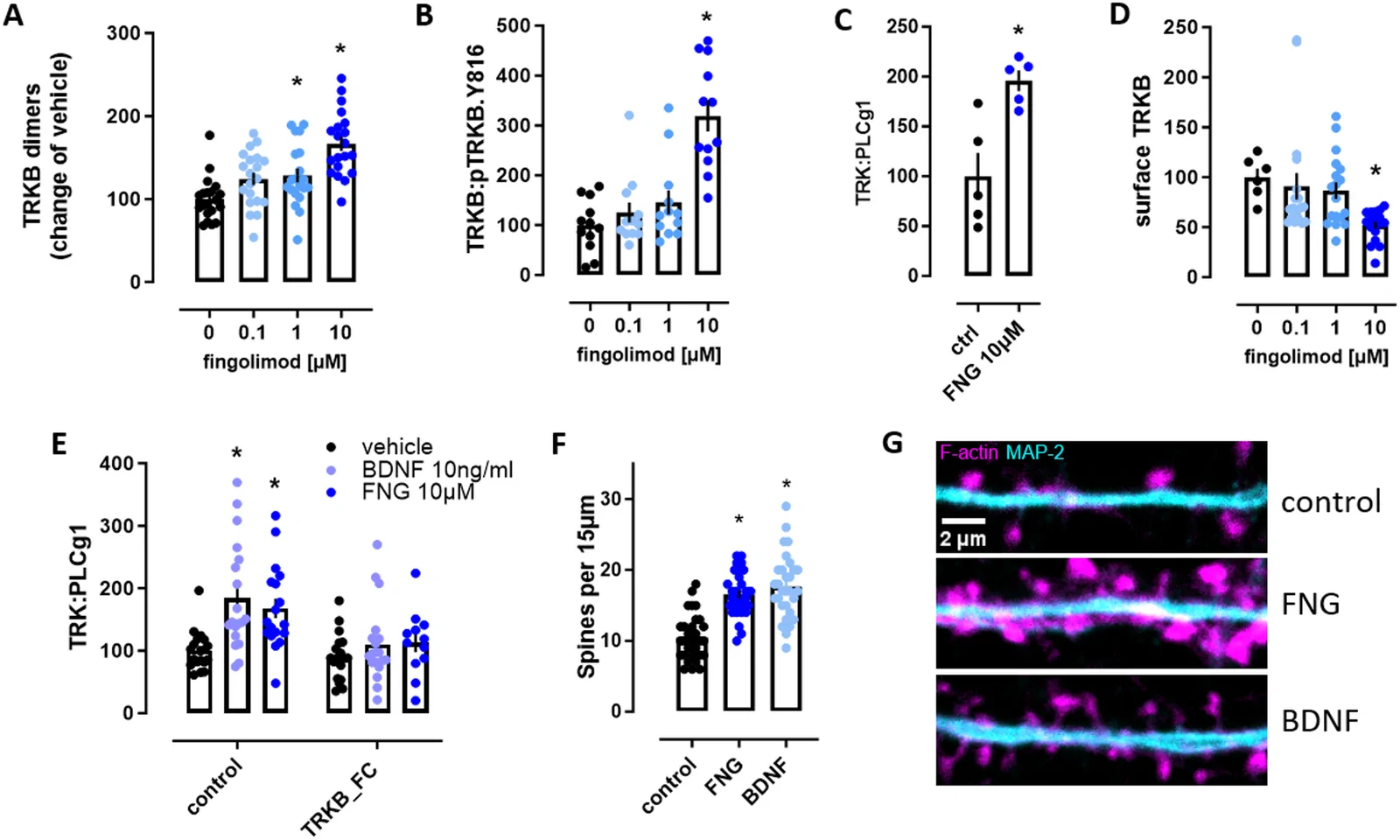
FNG induces TRKB signaling in vitro. **A** Acute administration of FNG (0.1, 1, 10 µM, 30 min) on N2A cells induces TRKB homodimerization (*n* = 20/group) in PCA. Statistical analysis performed by One-way ANOVA and Dunnett´s post hoc test, F (3, 76) = 13,05, p(1 µM) = 0.0289, p(10 µM) < 0.0001. **B** Acute administration of FNG and the effect on TRKB phosphorylation at tyrosine 816 measured by ELISA in primary cultured cortical cells from rat embryos (*n* = 12/group). One-way ANOVA and Dunnett’s post hoc test, F (3, 44) = 18,11, *p* < 0.0001. **C** Acute administration of 10 µM FNG on TRKB: PLCg1 interaction measured by ELISA (*n* = 5/group). Unpaired t-test, t = 3,734, df = 8, *p* = 0.0053. **D** Detection of plasma membrane localized TRKB following acute FNG treatment (*n* = 5/18/group). Kruskal-Wallis and Dunnett’s post hoc test, KW = 17,81, *p* = 0.0011. **E** Effect of the BDNF-blocking peptide TRKB_FC on the effects of acute FNG treatment in TRKB-PLCg1 interaction measured by ELISA (*n* = 18/group). Two-way ANOVA and Tukey’s post hoc test, F (1, 100) = 15,93 *p* = 0.0001 for the treatment effect. **F** Compared to control, FNG treatment (1 µM) promotes spine formation after 24 h, comparably to BDNF. One-way ANOVA and Dunnett´s post hoc test, F(2,87) = 32.58, p(FNG, BDNF) < 0.0001. *n* = 30 neurons/group. **G** Representative images for the spinogenesis experiment shown in panel** F**. Data is expressed as mean + SEM

While other studies have investigated the effects of FNG on BDNF and TrkB signaling after 24 h of administration (Deogracias et al. 2012; Patnaik et al. 2020), we have here focused on early effects of FNG on TrkB activity. We first assessed whether acute FNG treatments (0.1, 1, and 10 µM, 30 min) are able to induce TRKB homodimerization in N2A cells. We used a protein-protein interaction assay, where N2A cells were overexpressing TRKB tagged with a split luciferase that reconstitutes and becomes active if the two proteins interact. N2A cells do not express endogenous TRKB (Supplementary Fig. 1A), however express small levels of BDNF (Chen et al. 2017; Haapasalo et al. 1999). FNG dose-dependently induced TRKB dimerization, with a significant effect at 1 µM (Fig. 1A). Similarly, when we tested by ELISA on cortical primary neurons whether TRKB was phosphorylated as a result of its activation, we found that FNG dose-dependently increases TRKB phosphorylation at the Tyr 816 site and this effect reached significance, more than threefold compared to the vehicle-treated control, at 10 µM (Fig. 1B). Consistently, the interaction between TRKB and its signaling partner PLCg1 was doubled at 10 µM FNG in cortical neurons (Fig. 1C), indicating that TRKB was activated, phosphorylated, and that PLCg1 signaling cascade was initiated. The effects on TRKB mimic the effects elicited by TRKB natural ligand, BDNF (Supplementary Fig. 1B, C, D, E).

Next, we assessed whether FNG treatment affects TRKB localization on the plasma membrane on primary cortical cells. While the lowest concentration of FNG did not alter surface TRKB levels, 10 µM FNG significantly reduced the amount of TRKB receptor at the plasma membrane at 30 min after FNG stimulation (Fig. 1D). This is consistent with the behavior of the receptor which, upon activation, is internalized and sequestered from the cell surface (Harrington and Ginty 2013; Sommerfeld et al. 2000), further indicating that FNG promotes TRKB activity and signaling. We then investigated the role of BDNF on FNG-induced modulation of TRKB activity. We found that the interaction between TRKB and its signaling partner PLCg1 induced by FNG after 30 min treatment, was blocked when primary cortical neurons were pretreated with TRKB_FC, a TRKB-like peptide that sequesters extracellular BDNF impeding its binding to TRKB receptors (Fig. 1E), indicating that BDNF is required for the effects of FNG on TRKB signaling. To confirm that the observed early changes in TRKB activity and signaling are translated into long-term structural changes, we performed a spinogenesis experiment, which confirmed that, in line with previous evidence (Patnaik et al. 2020), FNG treatment promotes dendritic spine formation in hippocampal neurons comparably as the positive control BDNF (Fig. 1F-G).

**Fig. 2:**
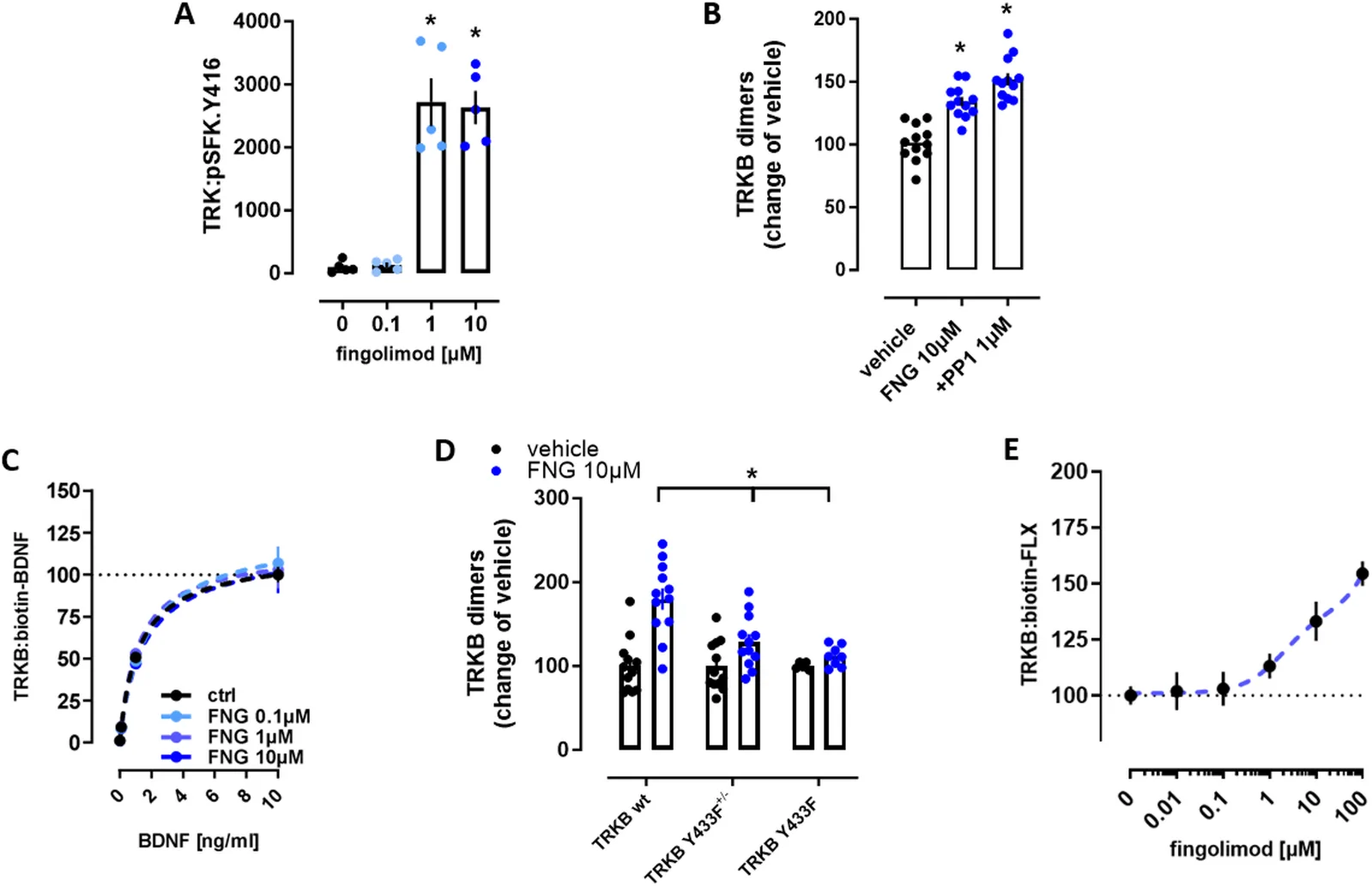
TRKB activation by FNG is an early event in FNG action and requires BDNF. **A** Acute administration of FNG and the activation of Src family kinase measured by ELISA on primary rat cortical neurons (*n* = 5/group). One way ANOVA and Dunnett´s post hoc test, F (3, 16) = 40,13, p(1 µM, 10 µM) < 0.0001. **B** Effect of acute FNG treatments on TKRB dimerization in N2A cells in presence of kinase inhibitors PP1 and k252a (*n* = 12/group). One way ANOVA and Dunnett´s post hoc test, F (2, 33) = 35,55, p(*) < 0.0001. **C** Ligand binding assay for TRKB and biotinylated BDNF in presence of increasing dose of FNG (*n* = 6/group). **D** Acute administration of FNG does not induce dimerization of the TRKB mutant Y433F (*n* = 12/group). Two-way ANOVA and Tukey´s post hoc test, F (2, 55) = 6,216, p(Y433F^+/−^) = 0.0028, p(Y433F) = 0.0002. Data is expressed as mean + SEM. **E** Effect of increasing concentration of FNG on the binding of biotinylated fluoxetine on TRKB (*n* = 6/group)

Because TRKB can be transactivated by other factors in absence of BDNF, in particular by Src family kinases (Huang and McNamara 2010; Rajagopal and Chao 2006), we assessed their involvement in the activation of TRKB following FNG treatment. Indeed, phosphorylation at Y416, an indication of Src kinases activation, was significantly increased after 30 min treatment with FNG (Fig. 2A). However, when we treated cells with the kinase inhibitor PP1 to inhibit Src family kinases (Hanke et al. 1996), we found that FNG was still able to promote TRKB dimerization, indicating that TRKB dimerization lays upstream of other cellular players that could contribute to TRKB activation and rather initiates a positive feedback loop of co-transactivation that is likely stimulated by BDNF binding to TRKB (Zhang et al. 2013) (Fig. 2B). Then, we performed a binding assay to investigate whether FNG treatment might promote the physiological interaction between BDNF and TRKB in cortical neuronal lysates. While we could detect an expected increase in TRKB binding with increasing concentration of BDNF, FNG treatment did not modify this binding at any concentration (Fig. 2C). This suggests that while BDNF is required for TRKB activation in response to FNG, the increased TRKB activity observed following FNG treatment does not occur by direct modulation of the binding between the receptor and its ligand.

TRKB contains in its transmembrane region an inverted cholesterol-recognition domain (CARC) that mediates its interaction with membrane cholesterol (Cannarozzo et al. 2021). We have previously observed that different classes of antidepressants, including psychedelics, can directly bind to the CARC domain of TRKB and promote its activity through BDNF (Cannarozzo et al. 2021; Moliner et al. 2023). We tested the dimerization induced by FNG on wild-type TRKB, and on a mutant TRKB (Y433F) that has been shown to be insensible to cholesterol binding (Casarotto et al. 2021) (Fig. 2D). Indeed, FNG induced dimerization of wild type TRKB after 30 min of exposure, but this dimerization was reduced both for wild-type/mutant couples and between two mutant molecules, indicating that the CARC domain and the transmembrane domain of TRKB are important for FNG-induced activity. We have previously shown that cholesterol promotes the binding of the antidepressant fluoxetine to TRKB, facilitating BDNF signaling, whereas antidepressants, which interact with TRKB dimers through the same domain as fluoxetine, compete with and thereby inhibit fluoxetine binding to TRKB (Casarotto et al. 2021). We therefore tested if FNG, due to its structural properties, might modulate TRKB activity by interacting with the CARC domain. Indeed, FNG increased fluoxetine binding to TRKB in a dose-dependent way (Fig. 2E), mimicking the effects of cholesterol but not that of antidepressant drugs (Casarotto et al. 2021).

### FNG Compensates for the Reduction of BDNF in Mice Exposed to Stress in Contextual Fear Conditioning

**Fig. 3:**
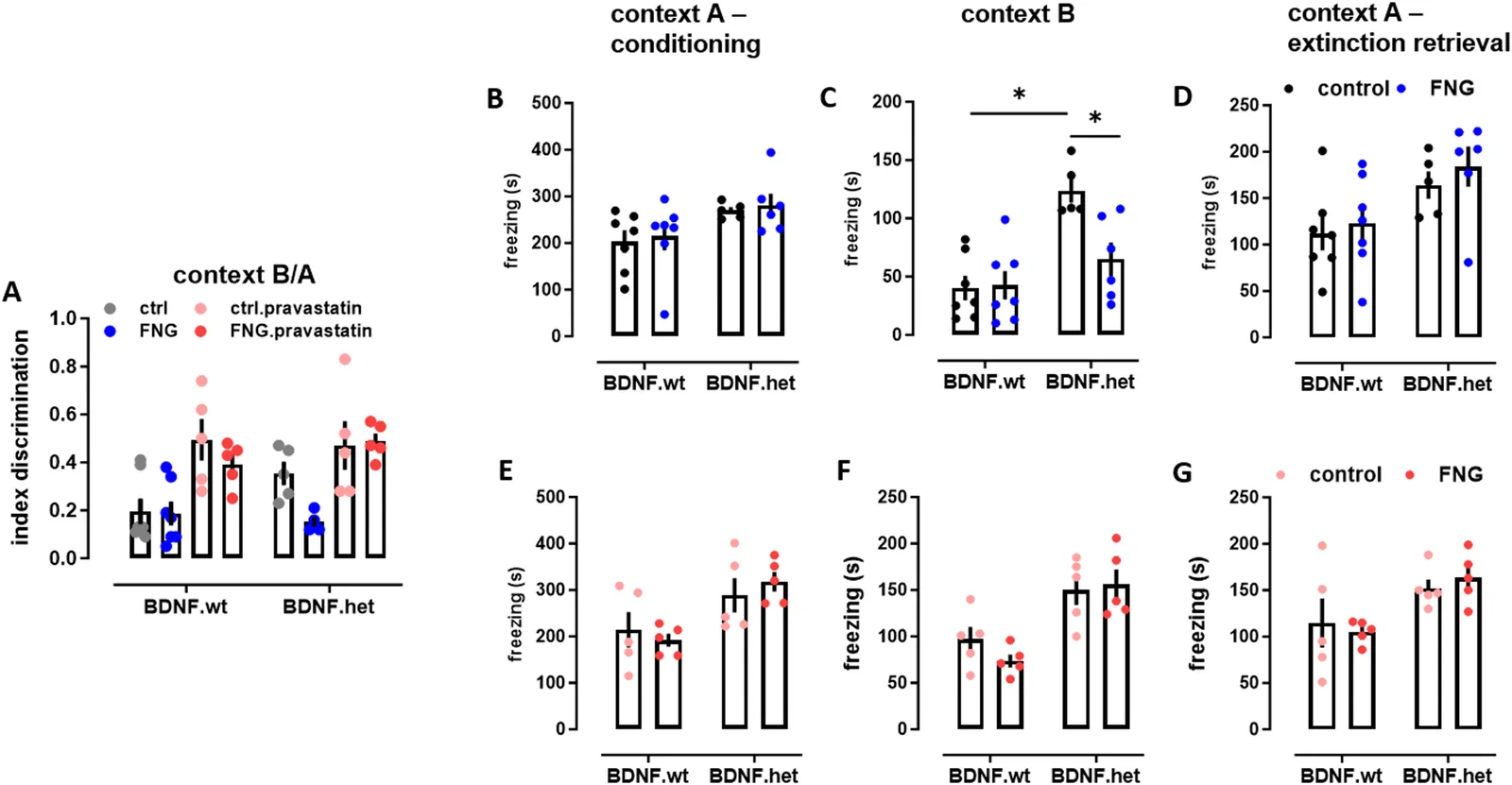
The effect of FNG administration on contextual fear conditioning. **A** Combined effects of pravastatin (10 mg/kg for 14 days in drinking water) and fingolimod (1 mg/kg, 2 h prior) on the ability of female mice to discriminate between context A and context B, expressed as discrimination index. Pravastatin treatment impairs the discrimination ability of the two contexts, Three-way ANOVA, main effect of pravastatin, F(1, 35) = 30,66, *p* < 0.0001. **B** 10 min foot shock conditioning session for BDNF.wt and BDNF.het mice. FNG treatment (1 mg/kg, 2 h prior) did not affect the freezing behaviour, Two-way ANOVA, F(1, 21) = 9,068, *p* = 0,9925 as main effect of the drug, while BDNF.hets generally freeze more compared to control, Two-way ANOVA F(1, 21) = 12.78, *p* = 0.0018 as main effect of the genotype. **C** FNG treatment (1 mg/kg, 2 h prior, blue bars) had no effect on BDNF.wt animals exposed for 5 min to a novel environment but could rescue the generalized freezing of the BDNF.het animals. F(1, 21) = 6,279, p(interaction) = 0,0205, p(controls) = 0.0006, p(BDNF.het) = 0.0199 by Two-way ANOVA and Tukey´s multiple comparisons **D** FNG treatment did not have any effect on the freezing behavior of BDNF.wt and BDNF.het mice placed back in the conditioning environment without foot shocks. Two-way ANOVA, F (1, 21) = 0,05193, *p* = 0,8219. Similarly as the conditioning, BDNF.het freeze more compared to wild-type littermates F(1, 21) = 8.636, p = *p* = 0.0078 for the genotype effect. In a cohort of animals pre-treated with pravastatin (10 mg/kg for 14 days in drinking water) (Panels **E**, **F**, **G**) FNG (1 mg/kg, 2 h prior) did not affect the conditioning. BDNF.het mice freeze significantly more compared to control animals (**E**), Two-way ANOVA F(1,16) = 11.8, *p* = 0.0034 for the genotype effect. **F** Similarly as for the condition, BDNF.het mice freeze more than the wt littermates, Two-way ANOVA, F(1,16) = 24.49; *p* = 0.0001 for the genotype effect. FNG treatment (1 mg/kg, 2 h prior) could not rescue the freezing behaviour in BDNF.het mice and had no significant effects on BDNF.wt animals. **G** Animals placed back in the conditioning environment without foot shock did not show any behavioral effect after FNG treatment (1 mg/kg, 2 h prior). As in the previous conditions, BDNF.het mice generally freeze more than the wt, Two-way ANOVA, F(1,16) = 9.374, *p* = 0.0075 for the genotype effect. Data expressed as mean + SEM, *n* = 5/7 per group

We next investigated the role of FNG in facilitating TRKB activity and whether FNG can mimic the effects of cholesterol in vivo. We tested whether FNG injections in wild type mice and mice lacking one allele of the Bdnf gene (BDNF.het) affected behavior in a contextual fear conditioning paradigm, with and without pravastatin pretreatment to deplete endogenous cholesterol. Cholesterol depletion by pravastatin (10 mg/kg, 14 days in drinking water) has been shown to affect TRKB signaling and downstream neuroplasticity in vitro (Suzuki et al. 2004) and in vivo (Casarotto et al. 2021). In line with previous evidence, in our experimental conditions, pravastatin worsen the ability of the mice to discriminate between two separate contextual environments (Fig. 3A). Both genotypes acquired conditioning, although BDNF.het mice showed elevated freezing. However, acute injections of FNG (1 mg/kg) two hours before being exposed to foot-shocks did not influence fear conditioning (context A) in either genotype (Fig. 3B). When exposed to a novel environment (context B) 24 h later, BDNF.het mice showed an elevated level of freezing, suggesting generalization of the fear response (Fig. 3C). FNG given 2 h before testing did not have any effects on wild type mice while it reduced the elevated freezing response in BDNF.het animals to a level seen in the wild-type mice. When the mice were returned to context A without being exposed to foot-shocks (extinction retrieval), BDNF.het mice again showed increased freezing when compared to wild type mice, but FNG treatment did not influence freezing in either genotype (Fig. 3D). These data suggest that while FNG has no effects in a physiological context after acute injection, it sensitizes TRKB signaling to BDNF in presence of reduced levels of BDNF. In pravastatin-treated animals, FNG treatment, although not statistically significantly, has a tendency to reduce freezing in the wild-type group while it did not rescue the freezing in BDNF.het animals (Fig. 3F). This suggests that FNG effects of BDNF sensitization on TRKB may partially mimic the effects of cholesterol, explaining why wild type animals with normal amount of brain cholesterol do not respond to FNG treatment. The fact that FNG could not completely rescue the reduction of cholesterol levels indicates that the effect of FNG and cholesterol on synaptic plasticity are not fully interchangeable, and cholesterol is still required for FNG action.

## Discussion

In this study, we identified an early mechanism for FNG, a sphingosine analog already approved for the treatment of MS, in modulating TRKB activity both in vitro and in vivo. We show that acute FNG treatment of neuronal cell lines and primary cortical neurons promotes TRKB activity in vitro within 30 min. Because there is no known mechanism for FNG to directly activate TRKB, and because the short treatment time may not allow for an increase in BDNF production, we hypothesized that FNG may facilitate BDNF binding to TRKB, acting as a positive allosteric modulator, and therefore promoting further activation, similarly to what was shown previously for other plasticity-inducing drugs (Casarotto et al. 2021; Moliner et al. 2023). Indeed, FNG-induced TRKB activation was BDNF dependent, indicating that FNG acts as an allosteric potentiator of TRKB, sensitizing it to the effects of BDNF. Increased BDNF expression has been implicated in the plasticity-promoting effects of FNG (Deogracias et al. 2012; Doi et al. 2013; Golan et al. 2019; Vidal-Martinez et al. 2019; Yu et al. 2023). However, it has been unclear how this effect is mediated. BDNF mRNA is regulated by neuronal activity and calcium entry is a central stimulus for the increased BDNF expression (Zafra et al. 1990). However, activation of TRKB receptors increases BDNF mRNA production in a positive feedback loop manner (Tuvikene et al. 2016; Yasuda et al. 2007). Previous studies have shown that ERK phosphorylation is increased in response to FNG stimulation within 30 min, whereas BDNF mRNA is increased after 60 min (Deogracias et al. 2012), indicating that TRKB activation may contribute to the increase in BDNF mRNA levels after FNG treatment. Non-neuronal cells can sense and produce BDNF, possibly mediating the effects of FNG on plasticity and behavior (Albini et al. 2023). Moreover, astrocytes regulate synaptic BDNF sequestering and release influencing synaptic plasticity (Vignoli et al. 2016), while recently gap junction-mediated astrocytic networks were shown to influence behavior (Cooper et al. 2026), indicating that the effect of drugs may not be limited to neuronal populations only. It was shown that FNG can upregulate production of neurotrophic factors, including BDNF, in activated microglia (Noda et al. 2013), but FNG treatment on isolated astrocytes failed to induce BDNF production (Lee et al. 2017). This suggests that the effect of the drug may be context dependent and preferentially occurs in pathological conditions. In fact, the primary therapeutic role of FNG on non-neuronal cells is via the sphingosine 1 phosphate receptor and induces downregulation of pro-inflammatory cytokines and is accompanied by improved neuroplasticity and production of BDNF (Choi et al. 2011; Groves et al. 2013; Miguez et al. 2015). Although it has been suggested that the effect on TRKB and BDNF are uncoupled from the effects on the sphingosine 1 phosphate receptor (Efstathopoulos et al. 2015), it is not possible to exclude a complementary role in the therapeutic effects of FNG, especially in a more complex in vivo context. In our study, we speculate that the acute effects of FNG administration are mediated by neuronal TRKB as astrocytes predominantly express the truncated form of TRKB that lacks the catalytic and signaling domain (TRKB.T1) (Rose et al. 2003), and the short treatment time likely does not rely on indirect mechanisms involving glial-neuronal crosstalk (Miguez et al. 2015; Noda et al. 2013).

Sphingosine (the physiological FNG analog) is constitutively present in the plasma membrane as a metabolite of sphingomyelin and ceramide and is known to induce membrane rigidity and order, similar to cholesterol (Alanko et al. 2005; Contreras et al. 2006). Together with other sphingolipids, it associates with cholesterol and is enriched in lipid rafts and synaptic membranes (Ikonen 2008). Moreover, chronic FNG treatment alters the lipid composition of the plasma membrane in the hippocampus of healthy young mice and accumulates in lipid-rich areas of the brain (Magalhães et al. 2024). This implies that FNG can insert into the lipid bilayer due to its apolar properties and induce the formation of ordered membrane microdomains, which are also known to be signaling hubs for TRKB (Pereira and Chao 2007; Suzuki et al. 2004). Investigating whether FNG modulates TRKB by altering membrane dynamics, we found that increased dimerization is prevented in TRKB (Y433F) mutants, suggesting that the effects of FNG are targeted to the transmembrane region of TRKB (Casarotto et al. 2021). FNG did not compete for TRKB interaction with the antidepressant fluoxetine, indicating that it does not bind to the antidepressant binding site within the TRKB dimer. In contrast, FNG promoted the binding of fluoxetine to TRKB in a manner that resembles the effects of cholesterol (Casarotto et al. 2021).

Our data suggest that FNG associates with the transmembrane domain of TRKB, thereby sensitizing TRKB to BDNF, which could then lead to increased production of BDNF mRNA and protein in a positive feedback fashion, as it was previously shown (Deogracias et al. 2012). In vivo, FNG could rescue the behavioral effects of decreased BDNF levels in contextual fear conditioning, a behavioral paradigm to assess hippocampal synaptic plasticity and memory (Phillips and LeDoux 1992). However, in mice with reduced overall cholesterol, which are already impaired in the tasks compared to controls, FNG is not able to rescue the behavioral effects anymore, indicating that FNG and cholesterol are not mutually interchangeable and physiological levels of cholesterol are still required for an effective FNG response. Observations by other groups of FNG effects on wild-type animals in similar behavioral paradigms could be explained by the long term administration of the drug (Efstathopoulos et al. 2015; Sun et al. 2016), remaining in line with the core findings of our study. We chose to assess the effect of FNG in a mouse model with generally reduced BDNF production, as the full knock out mice display reduced viability that would not allow for a proper interpretation of any behavioral outcome. Moreover, we show here that FNG effect requires the presence of BDNF as FNG alone is not able to activate TRKB in vitro, and the downstream effects are dependent on BDNF production (Efstathopoulos et al. 2015), therefore we can speculate that a full BDNF knock-out model would not be responsive to FNG treatment by increasing TRKB signaling. However, the use of conditional BDNF knock-out models (Monteggia et al. 2004, 2007; Taliaz et al. 2010) would provide useful information on the specific brain areas that preferentially mediate FNG activity, and whether sphingosine-1-phosphate receptor plays a role in the plastogenic effect of this drug.

Our data indicate that FNG administration sensitizes TRKB to the effect of BDNF early on both in vitro and in vivo, which may at least in part promote the increase in BDNF production observed the day after FNG treatment. Thus, we have identified an early action of FNG on TRKB that at least partially leads to the increased BDNF-mediated neuronal plasticity that is thought to underline many of the effects of FNG on neurological disorders.

## Supplementary Information

Below is the link to the electronic supplementary material.

Supplementary Material 1

## Data Availability

No datasets were generated or analysed during the current study.
