# Supplementary material for: Fingolimod Acutely Facilitates the Activation of TRKB in a BDNF-dependent Manner
Source: Cell Mol Neurobiol. 2026 Jul 8;46:145. doi: 10.1007/s10571-026-01774-y (PMC13631143; doi:10.1007/s10571-026-01774-y)
Supplement: Supplementary file 1 [file 10571_2026_1774_MOESM1_ESM.docx]

**Supplementary figure 1.** TRKB signaling *in vitro*, related to Figure 1. (A) Quantification of TRKB mRNA levels by qPCR in N2A cells non-transfected and transfected with TRKB.wt plasmid. (B) 30min BDNF treatment (10ng/ml) increase TRKB homodimerization by PCA in N2A cells. Unpaired t-test, t=8,351, df=30, p<0.0001, n=16. (C) BDNF (10ng/ml) increased TRKB phosphorylation at tyrosine 816 in cortical neurons in ELISA. Unpaired t-test, t=3,433, df=14, p=0.004, n=8. (D) BDNF (10ng/ml) increased interaction in cortical neurons between TRKB and its signaling partner PLCgamma1 in ELISA. Unpaired t-test, t=2,646, df=13, p=0.02, n=7/8. (E) BDNF (10ng/ml) promotes interaction between TRKB and activated Src family kinase in cortical neurons measured by ELISA. Unpaired t-test, t=2,512, df=12, p=0.027, n=6/8. Data is expressed as mean+SEM.
